# Novel Para-Aminobenzoic Acid Analogs and Their Potential Therapeutic Applications

**DOI:** 10.3390/biomedicines11102686

**Published:** 2023-09-30

**Authors:** Faisal Haroon, Umme Farwa, Maimoona Arif, Muhammad Asam Raza, Zeshan Ali Sandhu, Mohamed El Oirdi, Mohd Farhan, Mohammed Ahmed Ismail Alhasawi

**Affiliations:** 1 Department of Basic Sciences, Preparatory Year Deanship, King Faisal University, Al-Ahsa 31982, Saudi Arabia; meloirdi@kfu.edu.sa (M.E.O.); mfarhan@kfu.edu.sa (M.F.); 2 Department of Chemistry, Hafiz Hayat Campus, University of Gujrat, Gujrat 50700, Pakistan; ummefarwa025@gmail.com (U.F.); 21011707-006@uog.edu.pk (M.A.); zeshansandhu89@gmail.com (Z.A.S.); 3 Department of Medical Education, College of Medicine, King Faisal University, Al-Ahsa 31982, Saudi Arabia; 221408865@student.kfu.edu.sa

**Keywords:** para-aminobenzoic acid, antimicrobial, molecular target, synthesis, drug-like compounds

## Abstract

A “building block” is a key component that plays a substantial and critical function in the pharmaceutical research and development industry. Given its structural versatility and ability to undergo substitutions at both the amino and carboxyl groups, para-aminobenzoic acid (PABA) is a commonly used building block in pharmaceuticals. Therefore, it is great for the development of a wide range of novel molecules with potential medical applications. Anticancer, anti-Alzheimer’s, antibacterial, antiviral, antioxidant, and anti-inflammatory properties have been observed in PABA compounds, suggesting their potential as therapeutic agents in future clinical trials. PABA-based therapeutic chemicals as molecular targets and their usage in biological processes are the primary focus of this review study. PABA’s unique features make it a strong candidate for inclusion in a massive chemical database of molecules having drug-like effects. Based on the current literature, further investigation is needed to evaluate the safety and efficacy of PABA derivatives in clinical investigations and better understand the specific mechanism of action revealed by these compounds.

## 1. Introduction

**Figure 1:**
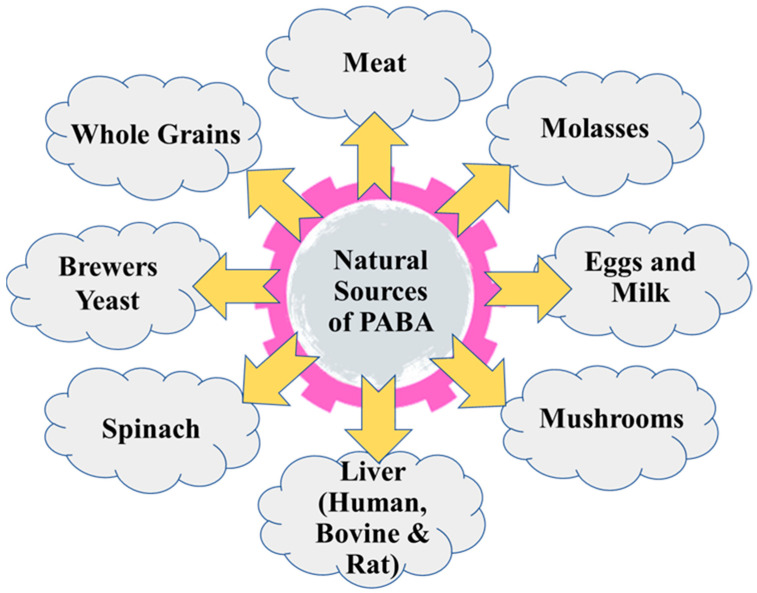
Representation of the natural sources of the *Para*-amino benzoic acid.

Aminobenzoic acids are compounds having carboxyl and amino groups directly bound to the aromatic ring [1]. Para-aminobenzoic acid (PABA) is an aromatic moiety having molecular formula C_7_H_7_NO_2_ [2], also well known as 4-aminobenzoic acid [3]. PABA has extensive usage in the chemical industry as a starting material for the preparation of folate, a crucial vitamin required for DNA synthesis and replication [4]. It is also used for the production of hair dyes and sunscreens due to its ability to absorb UV radiation [5]. Furthermore, the PABA is a non-toxic molecule, easily absorbed in the intestine, and its derivatives are capable of broad biological activities [6]. The drugs containing PABA scaffolds are believed to be well tolerated [7]. In folic acid synthesis, PABA is a necessary and irreplaceable vitamin of group B [8] and is found in various foods such as grains, milk, eggs, and meat. Through folic acid breakdown, it is also produced in the human body [9]. Although PABA is not synthesized in mammals and humans, it is a constant component of metabolism due to the supply of food and symbiotic bacteria (*Escherichia coli*), which continuously produce PABA in mammals [10]. The natural sources of *p*-aminobenzoic acid such as meat, egg, spinach, mushrooms, grains, etc., are presented in Figure 1. This review article covers the chemistry of *p*-aminobenzoic acid, chemical structural analogs of PABA, synthetic routes, and its biological potential as an anti-cholinesterase, antimicrobial, anticancerous, antiviral, and anti-inflammatory compound.

### 1.1. Chemistry of Para-Amino Benzoic Acid

**Figure 2:**
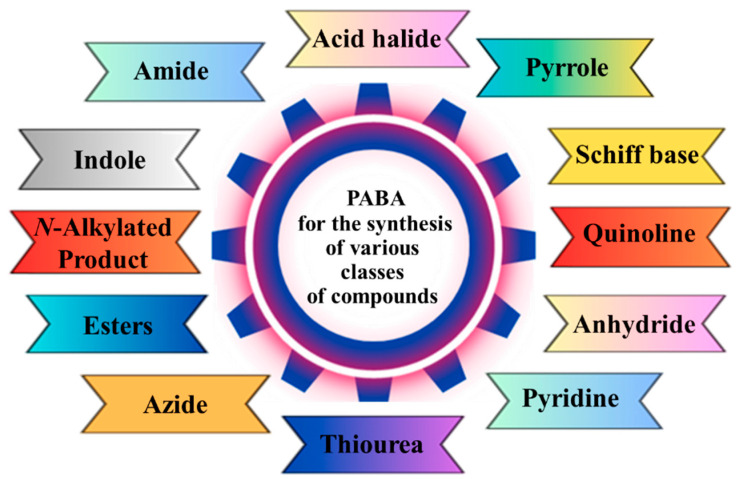
The synthetic derivatives of the *p*-aminobenzoic acid synthesized through different routes.

The chemistry of PABA involves the reactions of functional groups such as amino and carboxylic acid [11], while the amino group can undergo acylation with acetic anhydride to form an amide. The carboxyl group gives the esterification reaction with alcohol to form an ester [12]. PABA can also undergo oxidation with hydrogen peroxide or potassium permanganate to form 4-nitrobenzoic acid or 4-benzoquinone, respectively. Similarly, various moieties have been synthesized from PABA via different synthetic routes. The Schiff base, pyrrole, quinolone, pyridine, thiourea, indole, azide, etc., are the main compounds that have been prepared starting from PABA as shown in Figure 2. PABA can form coordination complexes with metal ions such as zinc as well as copper [13], and these complexes also have exhibited antibacterial action [14]. Overall, the chemistry of PABA is diverse and plays a crucial role in various biological and industrial processes [15]. Primary compounds of this group are unsubstituted isomers of aminobenzoic acid such as *m*-aminobenzoic acid, anthranilic acid, and *p*-aminobenzoic acid [16] and their derivatives containing halogen, aliphatic, or aromatic substitutions, which are being used widely in industry and chemistry. PABA has been known as a chemical compound since 1863 and as a drug with vitamin properties since 1939 [17]. Different substitutions at the *ortho*, *para*, and *meta* positions of the aromatic ring in PABA can lead to large variations in its chemical nature [18]. The chief factors that show a significant role in the chemistry of PABA are the inductive effect, mesomeric effect, and charge distribution [6]. PABA and esterified derivatives are well-thought-out classic examples of compounds that provide two protonation sites via carboxyl and amino groups [19]. PABA is also a biologically active compound that is frequently found as a building block for drugs with usages ranging from antimicrobial to UV protectants.

### 1.2. p-Aminobenzoic Acid as a Biological Scaffold

**Figure 3:**
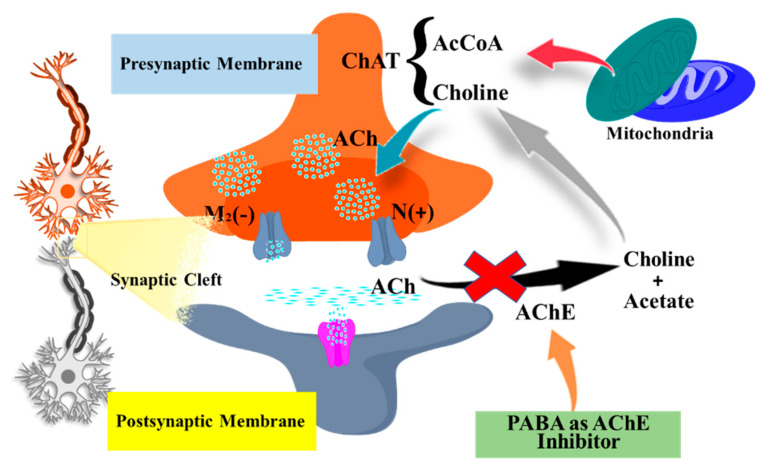
Mode of the action of the *p*-aminobenzoic acid during the inhibition of the acetylcholine esterase on synaptic cleft (ACh: acetylcholine; PABA: *para*-aminobenzoic acid; AChE: acetylcholine esterase; ChAT: choline acetyltransferase).

Alzheimer’s disease (AD) is dominant because of neuro disorders [20]. It is known as a chronic progressive neurodegenerative disorder that affects cognitive processes and intellectual abilities adversely [21]. For cognitive function, acetylcholine (ACh) is an important neurotransmitter, so enhancing acetylcholine levels at synapses is the primary treatment [22]. Recently, PABA has been featured as an important inhibitor of cholinesterase [23]. Several *p*-aminobenzoic acid derivatives have been evaluated against acetylcholinesterase (AChE) for AD treatment. Moreover, drugs containing PABA moiety are safe and tolerable [24]. The evaluation of several derivatives of *p-* and *m-*aminobenzoic acid as inhibitors of cholinesterase suggested the greater activity of *p-*substitution derivatives than other analogs [25]. Several imides and amides of *p-*aminobenzoic acid have been evaluated against AChE activity, which suggested that Schiff bases could be an encouraging moiety to treat learning as well as memory [26]. Figure 3 depicts the mechanism of the inhibition of AChE. AChE hydrolyzes the Ach into acetate and choline, due to which the message at the receptor cannot be received. The choline is again converted into ACh by reacting with AcCoA by catalyzing ChAT.

Numerous studies revealed that drugs incorporating PABA have shown diverse therapeutic effects, including local anesthetic, anti-tuberculosis, anti-convulsant, and anti-neoplastic effects. Furthermore, PABA is also included in peptidomimetic drugs. According to statistical analysis results, it is included in more than 184 drugs in the database of commercial drugs, and it is considered a building block in drug design [27]. During the last decade, prominent pathogenic bacterial and parasitic resistance has been observed toward chemotherapeutics in the market [28]. A serious concern is needed in the development of novel chemotherapeutic agents to compete with multi-drug-resistant strains [29]. Sulfonamides, which are the mimetics of *p*-aminobenzoic acid (PABA), led the antibacterial market for 50 years. The vast spectrum of biological activity of derivatives of PABA makes it attractive for developing novel antimicrobial agents [30]. In combination with some antibiotics, PABA, as opposed to being important for some bacteria, applies synergistic antibacterial potency to different strains including Pseudomonas aeruginosa and Staphylococcus aureus, although PABA alone also exerts antibacterial activity [31].

**Figure 4:**
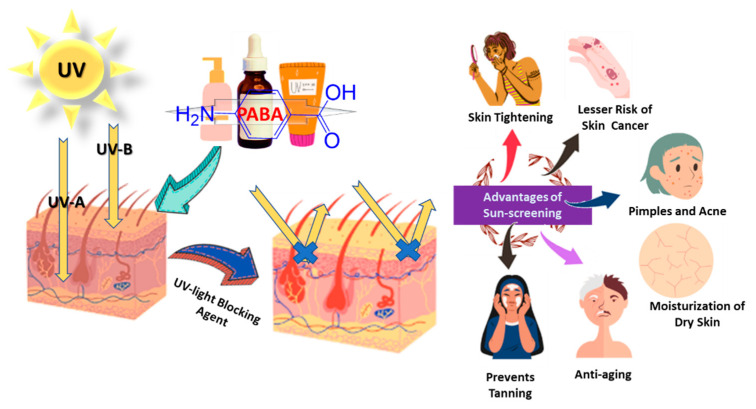
Representation of *p*-Aminobenzoic acid as a sun-screening agent for human beings.

After cardiovascular diseases, cancer is reviewed as the second most prominent reason for death [32]. Targeted chemotherapeutical agents are favorable over traditional agents because of their lesser side effects and selectivity towards cancerous cells [33]. Most commonly, DHFR inhibitors are conventional anti-folates, including pemetrexed (PMX), pralatrexate, methotrexate (MTX), and aminopterin. The chemical structure of the MTX prototype mainly consists of three significant moieties including *p*-amino benzoic acid, glutamic acid, and pteridine nucleus [34]. Some 1,2,4-triazoloquinazolines were found effective as antioxidant, anti-diabetic, antimicrobial, anti-hypertensive, and anti-inflammatory compounds [35]. The 5-chloro-[1,2,4]-triazolo quinazolines exhibited significant cytotoxicity toward cancer cell lines. *p*-Aminobenzoic acid (PABA), which belongs to the vitamin B group, is one of the pharmaceutically related small organic molecules that possess numerous biological applications [36]. The general sun-screening process is given in Figure 4.

## 2. Derivatization of *p*-Aminobenzoic Acids

**Figure 5:**
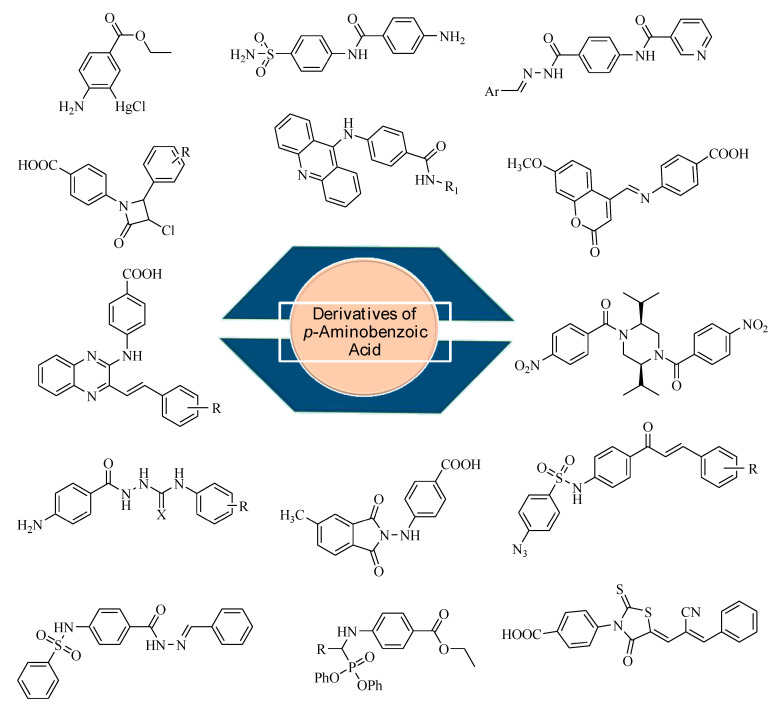
The reported derivatives of *p*-aminobenzoic acid having diverse functionalities (adapted from refs. [6,11,38,39,40,41,42,43,44,45,46,47,48,49]).

All of the derivatives that are presented with their structures in Figure 5 are used for different purposes in various applications. The chromophore of all of these compounds is *para*-aminobenzoic acid. It is clear from Figure 5 that such compounds having diverse functionalities have been prepared from PABA. Derivatives of PABA were synthesized as 1, 3, 5-triazine [37], quinoxaline [38], thiosemicarbazides, semicarbazides [39], sulfonamide [40,41], acridine [42], organometallic-based compounds [6], phthalimide [43], α-amino phosphonates [44], nicotinamide [45], a coumarin-based Schiff base [46], *p-*aminobenzoic acid-A [11], *p*-nitrobenzoic acid [47], new azidosulfonamide chalcones [48], and rhodanine [49].

### 2.1. Synthesis of Anti-Cholinesterase Agents

**Scheme 1:**
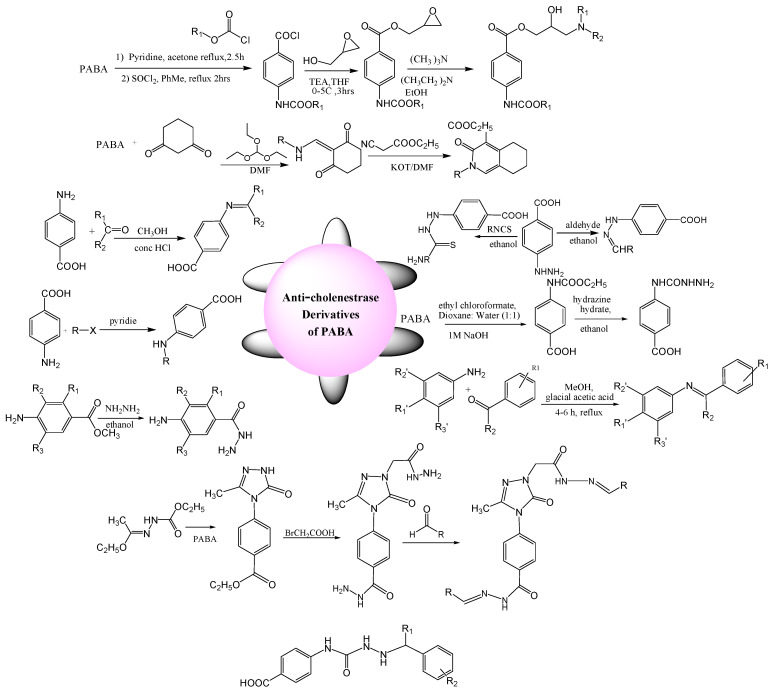
Synthetic routes for derivatives of *p*-aminobenzoic acid having acetylcholinesterase inhibition (adapted from refs. [23,25,26,50,51,52,53,54,55,56]).

A series of aminobenzoic acids derivatives were synthesized by the addition of the amine-based moieties as nucleophiles to the carbonyl group, resulting in the formation of an unstable amino methanol intermediate, which, in acidic conditions, undergoes dehydration to synthesize an imine [26]. Through Mannich three-component synthesis, methylene-substituted benzoic acid analogs were synthesized at 100 °C. The end products were experimentally assessed for in vitro action against AChE and CA, and in silico validation was performed with some software [50]. Schiff bases of para-aminobenzoic acid were prepared through the reaction of *p*-aminobenzoic acid with reported aldehydes and ketones using HCl and methanol. Evaluation of these Schiff bases for cognition-enhancing activities was performed through AChE inhibition [51]. Derivatives of 2-, 3-, and 4-aminobenzoic acid were synthesized by adding aromatic halides to the stirred solution of aminobenzoic acids in pyridine. For benzyl-based derivatives, the reaction mixture was refluxed for more than 24 h [52]. 4-aminobenzohydrazide compounds were synthesized from 4-aminobenzoate by reacting with hydrazine in the presence of ethanol under reflux [53]. Hydrazine carboxamido benzoic acid derivatives were synthesized from the carbamate ester of PABA that was reacted with formate in alkaline conditions in the solution of 1,4-dioxane and water to form ethoxycarbonylamino benzoic acid. The synthesized compound on further reaction gave hydrazine carboxamide benzoic acid derivatives [25]. Hydrazine carboxamide benzoic acid derivatives were prepared as presented in Scheme 1 [23]. Derivatives of Schiff bases were synthesized as aminobenzoic acid while ethyl ester was prepared by reacting with carboxylate derivatives under the microwave irradiation synthesis method to yield benzoate, which, upon further reaction with the bromoacetic acid, resulted in triazolyl benzoate derivatives. To attain modifications, products with dual functionality were obtained in the presence of hydrazine hydrate [54]. An equal amount of pyridine was added to the 4-aminobenzoic acid solution in acetone. To obtain the carbamate intermediates, suitable substituted chloroformate was dropwise added; the reaction mixture was heated at 70 °C; then, thionyl chloride was added to obtain acyl chloride intermediates; and later on, oxirane intermediates were prepared. Finally, tertiary amines and quaternary ammonium salts were prepared [55] as shown in Scheme 1. 6-aryl methyl ketones underwent condensation with DMF and DMA given enaminones that, consequently upon reaction with aminobenzoic acids, yielded targeted acids. Through the Mannich reaction, acetophenone was converted to obtain phenylpropanone derivatives, which were further converted to targeted carboxylic acid [56]. Scheme 1 represents the protocols for the synthesis of different derivatives of PABA for the acetylcholinesterase inhibitor.

### 2.2. Anti-Cholinesterases *Activity*

The enzyme inhibition assessment of PABA-derived compounds was performed against acetylcholinesterase (AChE) and butyrylcholinesterase (BChE), and the IC_50_ was also determined compared to standard donepezil. The studies showed that dimethoxy-*N*-(2,3,4-trimethoxybenzylidene)aniline is a potent inhibitor against AChE [26]. The results of AChE activity revealed that the *K_i_* value ranges between 13.62 ± 0.21 and 33.00 ± 0.29 nM in comparison with TAC for AD treatment [50]. An in vitro study exhibited maximum activity of 4-((bis(4-hydroxyphenyl)methylene)amino)benzoic acid with IC_50_ values of 7.49 ± 0.16 µM compared to rivastigmine, whereas *K_i_* was 8.14 ± 0.65 for AChE inhibition. Compounds were also biologically screened against the cholinesterase enzyme [51]. The cholinesterase enzyme inhibition assay showed that benzylaminobenzoic acid has maximum inhibitory activity with the lowest IC_50_ 2.67 ± 0.05 µM against BChE. Molecular modeling studies predicted good binding energy (∆G = −5.53 Kcal mol^−1^) [52]. The inhibitory potential of mono- or di-substituted benzohydrazide moieties was investigated against AChE and BChE. According to the results, 4-amino-3-bromo-5-fluorobenzohydrazide inhibited AChE and BChE and showed an IC_50_ value and binding affinity of 0.59 and 0.15 μM and −7.3 kcal/mol and −6.8 kcal/mol, respectively [53]. The *K_i_* values of three synthesized compounds indicated the greatest inhibition effect against AChE and BChE with values ranging from 0.10 ± 0.04 to 5.10 ± 2.14 μM. An in vitro enzyme kinetic study exhibited carboxamide-based derivatives as a non-competitive inhibitor of AChE and BChE with values of, respectively, *K_i_* = 0.041 ± 0.60 and 8.46 ± 0.66, which is comparable to donepezil [25]. To inspect the SARs for a series of *p*-aminobenzoic acid derivatives with AChE inhibition potency, 3D-QSAR in combination with molecular docking was employed. The hCA II inhibitor in all analog series having an alkoxy group was distinguished by an IC_50_ of 0.0514 μM. Derivatives of PABA had significant therapeutical potential for inhibiting the acetylcholinesterase enzyme [55]. PABA derivatives produced the maximum inhibitory activities against hCA [56].

## 3. Synthesis of Antimicrobial Agents

**Scheme 2:**
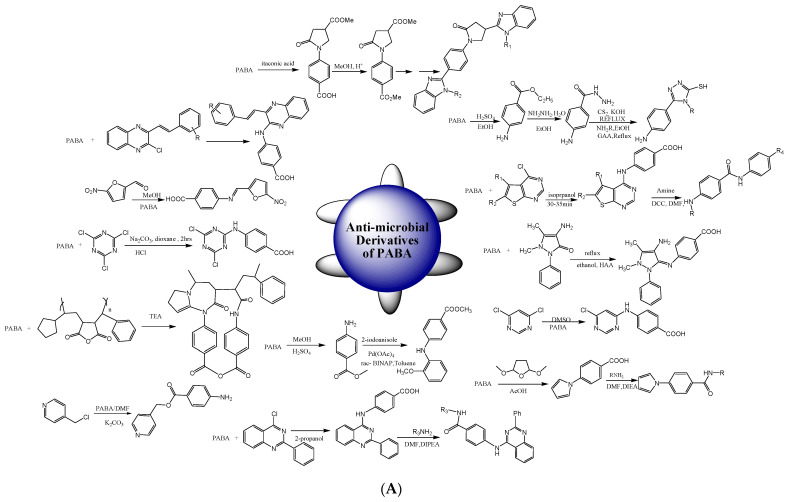

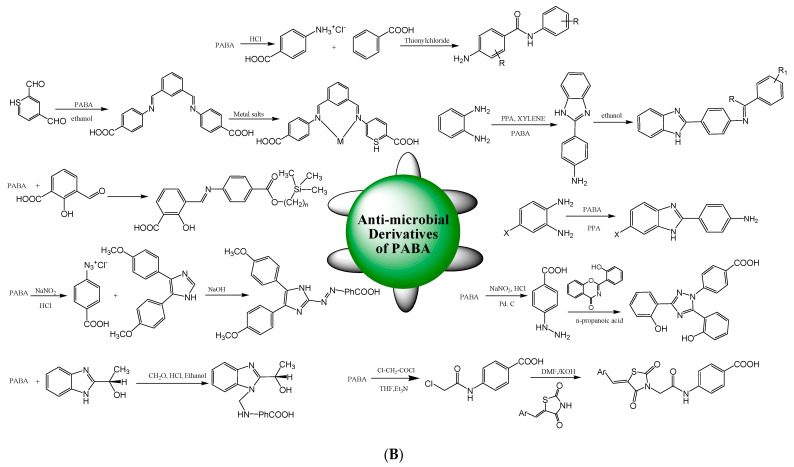
(**A**): Synthetic routes for derivatives of *p*-aminobenzoic acid having antimicrobial potential (adapted from refs. [30,31,38,57,58,59,60,61,62,63,65,66]). (**B**): Synthetic routes of derivatives of *p*-aminobenzoic acid having antimicrobial potential (adapted from refs. [69,70,71,74,75,76,77,79,80]).

The preparation of triazole-3-thiol moieties from PABA was carried out using green synthesis methodology. 4-amino benzohydrazide undergoes reflux with carbon disulfide in the presence of alcoholic potassium hydroxide to synthesize oxadiazole-2-thiol derivatives [57] as presented in Scheme 2A. Novel pyrimidines were also synthesized through the microwave assistance method. Firstly, 2-amino-3-carbethoxy thiophenes were prepared by employing a Gewald reaction, which then underwent cyclization with formamide and upon chlorination yielded the end products. Then, nucleophilic displacement of chloride with appropriate amines led to the desired thienopyrimidines [58]. Pyrrolidine derivatives from *p-*aminobenzoic acid were also prepared using azole, oxadiazole, benzimidazole, triazole, dithiosemicarbazide, and dihydrazone. The oxo-pyrrolidine was synthesized using itaconic and aminobenzoic acids that, upon esterification, gave methyl ester, which further reacted with hydrazine to give hydrazide. Chemical transformations of hydrazide were performed by applying different carbonyl compounds [30]. The synthesis of quinoxaline derivatives was carried out using *p*-amino benzoic acid, 2-chloro-3 substituted styryl quinoxaline, and triethylamine and refluxing in ethanol for 11 h [38]. PABA-derived Schiff bases were synthesized by dissolving 4-aminobenzoic acid in methanol, and, after that, one portion of the reported aldehyde was added, refluxed for 3 h, and then stirred at room temperature for 12 h [31]. Triazine and methyl ester from aminobenzoic acid analogs were synthesized [59]. The esterified products of *p*-substituted nitro and amino benzoic acid were synthesized in the presence of potassium carbonate and dimethylformamide by the *o*-alkylation of the COOH group, followed by alkylation of *p*-nitrophenol and *p*-acetaminophen [60].

The synthesis of carboxamides and rhodamine-based carbazole was performed. Firstly, Buchwald–Hartwig coupling was achieved through the reaction of the substituted aminobenzoic acid with 2-iodoanisole to produce methyl 4-(2-methoxyphenylamino)benzoate, which was converted into carbazole carboxylate derivatives after being treated with copper and palladium salt in glacial acetic acid (Scheme 2A). The resulting product was subjected to further treatment with suitable substituted alkyl and aryl amines in trimethylaluminum to obtain the targeted carbazole carboxamides, which, upon hydrolysis in alkaline media, resulted in carbazole carboxylic acid. The final product was obtained upon treatment with substituted primary and secondary amines using a coupling reagent to synthesize 8-Methoxy-*N*-substituted-9*H*-carbazole-3-carboxamides [61]. With the use of an AIBN initiator in acetone, terpolymer was prepared, which was converted to 2,4-dichlorophenol for introducing antibiotic activity [62]. Innovative various Schiff bases and their metal complexes were synthesized through the condensation of 4-amino anti-pyrine with 2,3 and 4-aminobenzoic acid by using a conventional method [63]. New zinc-based 4-aminobenzoate complex compounds were synthesized by adding an aqueous solution of ZnCl_2_ to an aqueous solution of Na_2_CO_3._ Then, from NaCl, fresh ZnCO_3_ precipitate was purified, a water suspension was added to the methanol solution of 4-aminobenzoic acid, stirred, and, later on, filtration, refluxing, and drying of the product was performed [64]. Benzamide derivatives were also prepared, and its chlorinated derivative, upon treatment with aminobenzoic acid, yielded quinazolin-4-yl amino benzoic acid, while amides were formed with different substituted amines [65]. Pyrrolyl benzamide derivatives were prepared using a pyrol ring and amino group of 4-aminobenzoic acid. Targeted compounds were also prepared through the reaction of pyrrolyl benzoic acid with substituted aromatic amines utilizing peptide-forming agents [66].

The Pf-DHFR inhibitors were developed using PABA and substituted aminopyrmidine. In silico screened compounds were also synthesized via the microwave assistance method [67]. The design and synthesis of aminobenzoic acid derivatives was achieved by reacting 4-amino benzoic acid and hydrochloride and refluxing [68]. The Benzimidazolyl benzamine compound was firstly synthesized from PABA and *o*-phenylene diamine, which, after further reacting with formaldehyde and piperidine, resulted in a piperidinylmethyl-based benzamine derivative [69]. Azo-based derivatives of already reported compounds were synthesized by reacting a solution of sodium salt and calixarene, followed by the addition of the diazonium salt of PABA [70]. From the condensation of 3-formyl salicylic acid with trimethylsilyl-propyl-paminobenzoate and trimethylsilyl-methyl-*p*-aminobenzoate, two Schiff bases were derived [71]. Azo ligand and its metal complexes were synthesized by coupling methoxy phenylimidazole and the diazonium salt of PABA at 0–5 °C, and complexes were prepared by reacting the ligand with metal salts [72]. Through the reaction of diazonium salt of *p*-aminobenzoic acid and pyrogallol, the azo reagent was prepared, and three chelate complexes with the metal ions of Co, Ag, and Ni were also obtained [73].

The three-component synthesis of new chiral benzimidazole Mannich bases was performed by a reaction between benzimidazole, 30% formaldehyde in water, and an amine [74]. The synthesis of *o*-substituted benzimidazole with imine linkage-based compounds was also performed. Firstly, condensation of *o*-phenylenediamine with PABA in xylene and polyphosphoric acid resulted in aminobenzimidazole, which was further treated with substituted aldehydes and ketones [75]. Metal complexes of cobalt, copper, nickel, cadmium, and zinc were also synthesized. Two Schiff bases were derived by condensing *m*-pthalaldehyde with 4-aminobenzoic acid and isonicotinic hydrazide, which were added to the methanol solution of CuNO_2,_ ZnNO_2_, CoNO_2_, NiNO_2_, and CdNO_2_ [76]. A 1,2,4-triazolebenzoic acid moiety was synthesized by starting with toluene to obtain 4-hydrazinobenzoic acid, and then condensation of substituted benzoic acid resulted in the target compound [77]. Nano-sized cadmium oxide and manganese oxide were formed via chemical pathways, using the calcination from an aqueous solution that created metal chloride to create polyvinyl alcohol and 4-aminobenzoic acid complexes with Mn and Cd [78]. The Knoevenagel condensation method was also used for the reaction of aldehydes and thiazolidinedione to yield thiazolidinediones derivatives via microwave irradiation, and *N*-alkylation in the basic medium was performed to obtain the targeted products [79]. 2-phenylbenzimidazole compounds were synthesized by refluxing phenylenediamine and PABA in polyphosphoric acid up to 8 h at 220 °C (Scheme 2B). Benzimidazole/thiourea derivatives were achieved by reaction of the preliminary compounds with isothiocyanate and CH_2_Cl_2_ [80]. Metal complexes were synthesized by using a refluxing technique [81]. Triazolethiol derivatives were also synthesized in four steps by refluxing PABA in the presence of sulphuric acid to yield aminobenzoate derivatives, which, on further refluxing with hydrazine, yielded hydrazide derivatives. The synthesized moiety was further refluxed in alcoholic KOH with CS_2_ to form diazolethiol derivatives as shown in Scheme 2B. Further refluxing this compound with amine in ethanol gave triazolethiol as the end product [57]. Scheme 2A,B represented the protocols for the synthesis of derivatives of PABA having antimicrobial potential.

### Antimicrobial Activity

These compounds were screened for antimicrobial activity against *E. coli*, *S. aureus*, *B. subtilis*, *K. pneumonia*, and fungi, i.e., *S. cerevisiae* and *A. niger*. The antimicrobial evaluation identified that 4-chloro-6-nitro-, 6-chloro-, and 4-nitro-substituted benzothiazole-triazolethiol derivatives have significant potential as compared to Ketoconazole and Norfloxacin (standard drugs) [57]. Thienopyrimidinylamino-*N*-phenylbenzamide and its *N*-(4-chlorophenyl)-substituted compounds possess significant inhibitory potencies against *P. aeruginosa*, *S. aureus*, *B. subtilis*, and *E. coli,* with effective MICs (2–10 μg/mL) [58]. The targeted PABA derivative is more effective against *S. aureus* (methicillin-resistant), having an MIC value of 4 μg/mL. The microbial inhibitory potential was greater than that of ampicillin. Additionally, benzimidazoles revealed good antibacterial action with an MIC value of 15.62 µg/mL against *L. monocytogenes,* showing four times more potent results than ampicillin [30]. Among all of the compounds, cyanostyrylquinoxalinyl-based PABA was found to be the most active one for the inhibition of different strains, having an MIC in the range of 7.9–31 µM [38]. The antimicrobial activity and cytotoxicity of Schiff-based compounds were determined. The PABA was simply chemically modified, resulting in the establishment of antibacterial activity, including the inhibition of Staphylococcus aureus with an MIC found to be 15.62 µM, modest antibacterial potential of MIC ≥ 62.5 µM, and effective antifungal properties (MIC of ≥ 7.81 µM) [31]. The antimicrobial activity of triazine analogs along with their methyl esterified products was assessed. These compounds showed greater inhibitory potential against *E. coli* and *S. aureus* comparable to ampicillin [59]. The antibacterial properties of the synthesized compounds were greater toward Gram-negative than Gram-positives, and amino derivatives were more effective with MIC values of 0.16 mM [60]. Preliminary in vitro antibacterial and antifungal activity was evaluated for all PABA-derivatized compounds, which exhibited notable antifungal and antibacterial activity. Additionally, these moieties also exhibited remarkable antimicrobial activity with an MIC against *C. neoformans*, *C. albicans,* and *S. aureus* pf 6.25 μg/mL, 12.5 μg/mL, and 1.56 μg/mL, respectively [61]. Evaluation of the antibacterial activity of terpolymers exhibited considerable antibacterial inhibition, and the MIC was found to be 75–80 mg/mL. These polymers exhibited 90% bactericidal activity at 200 mg/mL [62]. The activity demonstrated that the mixed complexes exhibit worthwhile performance against bacterial strains and efficiency of complexes in the order of Ni > Cu > L. Zinc carboxylate compounds were tested against different microbes. The presence of Zinc in the complexes enhanced antimicrobial potential in contrast to free 4-aminobenzoic acid and ligands [64]. Among all synthesized compounds, nine compounds showed good inhibition for *M. tuberculosis,* having MIC values of 4–32 µg/mL [65]. The newly synthesized chemical moieties were screened to target the enoyl-ACP reductase enzyme, the key enzyme of *M. tuberculosis*. All of the pyrrolyl benzamide compounds were evaluated against *M. tuberculosis* H37Rv and InhA inhibition [66]. Aminochlorofluoro phenyl benzamide displayed 60% activity against *E. coli* and 90% against *S. aureus* comparable to Ciprofloxacin [68]. Screening results against anti-inflammatory, antifungal, and antibacterial activities concluded that alkoxybenzaldehyde substituted benzamine was a highly potent one [69]. PABA-derived analogs showed noticeable antifungal and antibacterial activities [61]. An MIC potential ranging between 0.97 to 62.5 μg/mL was observed for sulphanilamide, 2-methyl-4-aminobenzoic acid, and sulfaguanidine for bacterial strains [70]. Both compounds proved to be better inhibitors of fungi than caspoungin as the standard according to in vitro antimicrobial results [71]. The biological potential of the synthesized ligand and its Pd complex against Gram-positive and Gram-negative bacteria showed that the azo ligand revealed an extraordinary inhibition zone against *E. faecalis* and a modest inhibition zone against *E. colibacteria*, while higher and modest inhibition activity against *E. coli* and *K. pneumoniae* was shown by the Pd complex [72]. The in vitro antibacterial activity of all the synthesized compounds showed that the presence of saturated heterocycles from the amine molecule, morpholine, and 1-methyl piperazine results in enhanced biological activity in comparison with other aromatic amino equivalents such as 4-nitroamino-diphenylamino and 4-aminobenzoic acid [74]. The biological potential of the synthesized dye and its complexes toward Gram-positive and -negative bacteria displayed good to moderate efficiency. The antibacterial evaluation of the synthesized compounds against *E. coli* and *S. aureus* exhibited that benzylidene benzenamine and phenyl iminomethylphenol were found to be stronger antibacterial agents than Vancomycin as a standard drug [76]. Efficient results of dibenzoic acid and its cadmium [77] salt were observed for antimicrobial action against two bacterial strains. The screening for antibacterial and antifungal activity of the synthesized derivatives of triazole benzoic acid gave positive results compared with ciprofloxacin and miconazole [78].

Outstanding antifungal and antibacterial activities were demonstrated by synthesized CdO and MnO nanoparticles along with PABA. Molecular docking study and in vitro antimicrobial activity results revealed that compounds were more active against *E. coli* and *S. aureus*. A notable antifungal action was observed for *C. albicans* (ATCC 10231) with an over 60% action in comparison to the standard drug [80]. Antibacterial activity was noticed in most of the benzimidazole derivatives. The results of enzyme inhibitory activity against DNA gyrase (*E. coli*) and topoisomerase IV (*S. aureus*) exposed better binding and dual inhibitory activity against both enzymes. The best results were shown by substituted pyrazolidinedione with 89.3% inhibition and an IC_50_ value of 0.58 uM against targeted enzymes [81]. The antimicrobial screening suggested three most potent compounds with broad-spectrum activity as compared to standard drug ketoconazole and norfloxacin [57]. Greater bacteriostatic activities were observed for the complexes than for free ligands [82].

## 4. Synthesis of Anticancer Agents

**Scheme 3:**
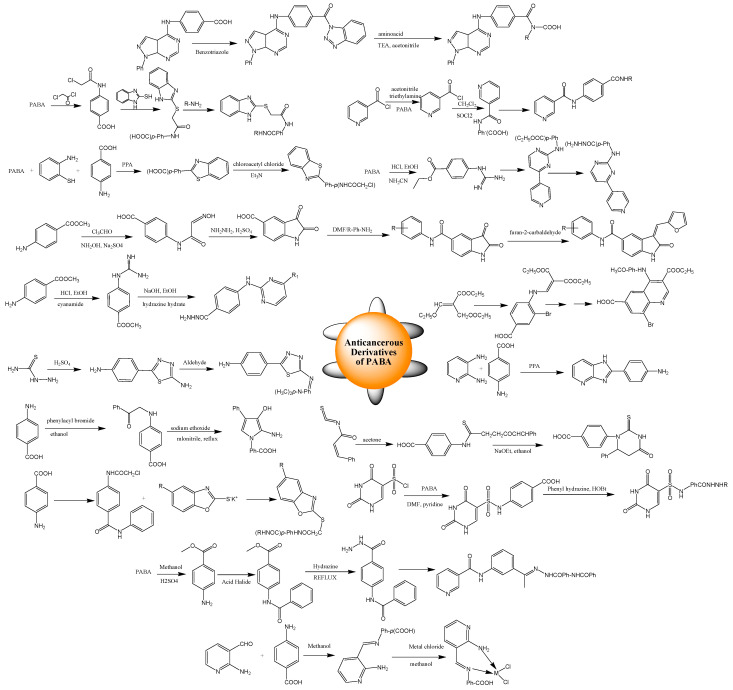
Synthetic routes of *p*-aminobenzoic acid derivatives having anticancer activities (adapted from refs. [33,34,83,84,85,86,87,89,90,91,92,93,95,96,97,101]).

PABA derivatives were obtained by reacting haloacetyl chloride with PABA, which was converted to benzo[*d*]imidazolylthio-acetamido benzoic acid by reaction with *o*-mercaptobenzimidazole; further, it was reacted with thionyl chloride, leading to the synthesis of benzo[*d*] imidazol-ylthio-acetamido-benzoyl chloride. This compound interacting with various substituted anilines in ethanolic solvent gave the desired products as presented in Scheme 3. Amino-methylthio-triazolyl benzoic acid and *p*-hydrazinobenzoic acid were derived from *p*-hydrazinobenzoic acid hydrochloride. *p*-Hydrazinobenzoic acid, upon reaction with different acid anhydrides, aldehydes, and isothiocyanates, yielded the end products. By starting with PABA, the following methylene oxoindoline carboxamide derivatives were yielded. Firstly, oxoindoline carboxylic acid and the Isatin compound were prepared by reacting PABA with hydroxylamine, trichloracetic aldehyde, and conc. sulfuric acid. The reduction of ketone was carried out, followed by amination with anilines using a coupling agent to form products, which, on coupling with pyrrole-carbaldehyde or furan-carbaldehyde, afforded the final compounds [83]. Aminophenyl benzothiazole was synthesized by reacting with PABA with *o*-aminothiophenol at 220 °C in polyphosphoric acid. The synthesis of benzo[d]thiazolyl-phenyl-chloroacetamide was achieved by treating aminophenyl-benzothiazole with haloacetyl chloride in the presence of triethylamine, and further reacting with piperazines-based compounds at 100 °C produced the end products. Benzo[d]thiazolyl-propynyl-aniline was synthesized by treating aminophenyl-benzothiazole with propargyl bromide [84].

The synthesis of amino-indole-carbohydrazide derivatives was achieved. Ethyl 4-guanidinobenzoate was obtained by refluxing ethyl-aminobenzoate and cyanamide. Again, we refluxed with compounds such as 2, 3, and 4-pyridyl-propenone. The resulting products reacted with hydrazine to form the reported products, which, followed by a condensation reaction with suitable aldehydes, gave hydrazone products [85]. Carboxamide and carbothioamide derivatives were yielded by first refluxing *p*-aminobenzoate with cyan-amide in the presence of conc. HCl and then treated with ammonium nitrate to synthesize 3 and 4-guanidino-benzoic acid ethyl ester nitrate. Further reaction with 3-(dimethylamino)-arylpropenones under reflux for 48 h afforded arylpyrimidinyl-amino benzoic acids, which, by again refluxing with hydrazine hydrate, was used to obtain the resultant aroylhydrzides. The final end products were synthesized by the reaction of aroylhydrzides with different isocyanates [86]. Compounds having several carbamoyl groups were obtained by starting from nicotinic acid. 4-(nicotinamido)benzoic acid was synthesized by an SNAr displacement reaction of the nicotinoyl chloride with PABA in TEA. *p*-nicotinamido-benzoic acid was further reacted with the thionyl chloride using dichloroethane to produce the compound, which, upon treatment with benzylamine, cyclohexylamine, and aniline analogs in acetonitrile, afforded the targeted compounds. Innovative pyrazolo [3,4-d]pyrimidines holding diverse amino acid conjugates were designed [87]. Phenyl-pyrazolo [3,4-d]pyrimidin-yl-aminobenzoic acid and key intermediates were prepared by reacting chloro-phenyl-pyrazolo [3,4-d]pyrimidine with PABA. These intermediates were further reacted with 1*H*-benzotriazole, giving the *N*-acyl benzotriazole compound, which, upon condensation with numerous amino acids, gave substituted amino acid compounds [34]. Pyrazolone compounds were achieved by condensing phenyl hydrazine derivatives with ethyl acetoacetate derivatives. Suzuki coupling between the derivatives of the pentagonal heterocyclic and benzene ring was performed to obtain intermediates that underwent Knoevenagel condensation to result in the desired compounds [88]. Quinoline derivatives were also produced from substituted amine through the Gould–Jacobs reaction and reaction of bromo-derived analogs with diethyl ethoxymethylenemalonate, resulting in the synthesis of enamine moieties. The cyclization of enamine derivatives in hot diphenyl ether, given the quinolone analogs and upon chlorination, yielded chloroquinoline products. Target 4-anlinoquinoline derivatives were obtained by nucleophilic substitution of chlorine with aromatic amines [89]. The synthesis of pyrrolopyrimidine and pyrrole moieties was achieved. Reacting 4-substituted amino benzoic acid with malononitrile under reflux in alcoholic sodium alkoxide yielded *p*-(2-amino-3-cyano-4-phenyl-1*H*-pyrrol-1-yl)benzoic acid [90]. *N*-benzoyl-(2,4-dihydroxypyrimidine-5-sulfonamido)benzoyl hydrazide derivatives and *N*-phenyl-(2,4-dihydroxypyrimidine-5-sulfonamido)benzoyl hydrazide derivatives were prepared. Aminobenzoic acid reacted with 2,4-dihydroxypyrimidine-5-sulfonylchloride in the presence of pyridine to synthesize the compound, which reacted serially with 1-ethyl-3-(3-dimethylaminopropyl)carbodiimide (EDCI) and hydroxybenzotriazole (HOBT) to obtain an ester that, upon reaction with the corresponding phenyl hydrazine, gave the desired products [91]. By condensation of 4-aminobenzoic acid with 2,3-diaminopyridine in polyphosphoric acid, 4-(1*H*-imidazo [4,5-*b*] pyridin-2-yl) aniline was obtained, which reacted with diethyl ethoxymethylenemalonate using absolute ethanol given the diethyl methylenemalonate derivative. The yielded compound was further treated with hydrazine hydrate in the presence of NaOEt, which yielded products. The resulting diester was refluxed in glacial acetic acid with hydrazine hydrate given products. Additional compounds were synthesized by reacting a diazonium salt-based compound with suitable phenolic compounds at 0–5 °C in 10% NaOH. The condensation of the diethylmalonate derivative with thiourea or urea synthesized thioxopyrimidinedione and pyrimidinetrione in NaOEt [33]. By condensing *N*-(4-acetylphenyl)nicotinamide with *N*-(4-(hydrazinecarbonyl)phenyl)benzamide, a nicotinamide-based derivative was designed as well as synthesized, where an imine group replaced the carbonyl group [92]. PABA derivatives such as benzoxazoles were also prepared. Potassium salts of 2-mercapto-benzoxazoles were prepared by reacting chloroacetylchloride with PABA to form acetamide, followed by an acylation reaction to synthesize 4-(2-chloroacetamido)benzoyl chloride, and, finally, the products were treated with amines in the presence of TEA. Heating of potassium salts of 2-mercapto-benzoxazoles in anhydrous DMF with the prepared derivatives was carried out to obtain the final compounds [93]. Compounds having core structures such as substituted arylamine, triazole, and coumarin were synthesized. The first intermediate was obtained through azide formation of PABA in NaN_3_/NaNO_2_ in water. The second intermediate was formed by substituting propargyl bromide in 4-hydroxycoumarin. These two intermediates were reacted with CuI in CH_2_Cl_2,_ yielding a compound, which, upon chlorination with SOCl_2,_ gave intermediates. The final compounds were obtained by reacting substituted arylamines with intermediates [94]. The complexes of the reported ligand of PABA with cobalt, copper, nickel, palladium, and zinc were synthesized. Ligand was synthesized by reacting 4-aminobenzoic acid with 2-aminonicotinaldehyde at stirring. Mixing metal chlorides with ligands yielded metal complexes in methanol [95] as shown in Scheme 3. Azoles and azines were formed by the reaction of PABA with isothiocyanate and heteroallene [96]. A thiadiazole-based compound was synthesized by the reaction of PABA with thiosemicarbazide upon refluxing for up to 7 h. Schiff bases were obtained through the reaction with dimethylaminobenzaldehyde at a reflux temperature for 6 h [97]. A series of quinazolinone derivatives were obtained: 3-(4-oxo-2-phenylquinazolin-3(4*H*)-yl)benzoic acid and 4-(4-oxo-2-phenyl quinazolin-3(4*H*)-yl)benzoic acid. 2-phenyl-4*H*-benzo[d][1,3]oxazin-4-one was refluxed with 4-amino benzoic acid pyridine, and further chemical variation of compounds gave the products [98]. Thiazolidinediones were prepared by starting with the condensation of chloroacetic acid with thiourea, which, upon reaction with benzaldehyde, resulted in mono and dichlorobenzylidene)thiazolidinedione products. On the other hand, chloroamide was obtained by reacting chloroacyl chloride with 4-aminobenzoic acid. The treatment of ethyl chloroformate with chloroamide and the addition of amine afforded the intermediate. The potassium salts were refluxed with these intermediates to form derivatives [99]. The final compounds were synthesized through the reaction of a hydrazone amide derivative with arylidene derivatives in ethanol under reflux. Thiazolidinone derivatives were obtained through the nucleophilic reaction between ammonium thiocyanate and chloroacetamide benzoate. Further refluxing this obtained product and malononitrile in an alcoholic solvent with drops of TEA afforded the resultant thiazole compounds [100]. Scheme 3 represents the protocols for the synthesis of derivatives of PABA having anticancer agents.

### Anticancer Activity

Anticancer results exhibited the minimum IC_50_ value for benzamide derivatives of PABA as 5.85 µM and 4.53 µM, and compounds exhibited higher anticancer activity than standard drug 5-Fluorouracil [101].

The cytotoxic action also demonstrated that many of the targeted compounds have strong inhibitory action against cell lines; MCF7 and HCT-116 had IC_50_ values ranging between 28.3 ± 5.1 µM and 21.3 ± 4.1 µM [35]. The three more potent compounds showed IC_50_ values comparable to doxorubicin. Furthermore, inhibition of the proliferation of MCF-7 was also depicted by some compounds. According to the primary biological test, significant inhibitory activity was observed against A549 cell line proliferation. The carboxamide derivative of PABA was revealed as the most potent one with an IC_50_ value of 3.0 μM [83]. Bio-evaluation of the antiproliferative activity of some of the synthesized molecules over three human cancer cell lines displayed considerable growth inhibition (GI_50_) in the range of 0.2–1.7 μM. Reasonable activity was exhibited by compound Benzo[d]thiazolyl)-methoxyphenyl-triazolyl methyl aniline against cancer cell lines with GI_50_ ranges from 0.55 to 1.2 μM [84]. Evaluation of synthetic compounds as CDK9 inhibitors exhibited exceptional antiproliferative activities of the compound amino-benzohydrazide against cancer cells with IC_50_ values ranging between 0.57 ± 0.02 µM and 1.73 ± 0.09 µM [85]. Among all of the synthesized structures, excellent antagonist activity was shown by compound *N*-butyl-2-(4-((4-(pyridin-4-yl)pyrimidin-2-yl)amino)benzoyl)hydrazine-1-carboxamide having EC_50_ = 1.68  ±  0.22 µM. Strong antiproliferative activity with IC_50_ values < 10 µM against human cancer cells was also indicated. Binding modes for potent compounds and RXRα-LBD were predicted using molecular docking studies [86]. Screening of all compounds against three cell lines evaluated the compound *N*-(4-((3-Methoxyphenyl) carbamoyl)phenyl) nicotinamide as the most active one with a noticeable in vitro VEGFR-2 inhibitory effect. A considerable rise in the caspase-3 level by a 7.80-fold 87% reduction in TNF-*α* was also observed [87]. The enzymatic inhibition screening of compounds against DDHFR and six MTX-resistant cancer cell lines indicated the 5-Guanidino phenyl pyrazolopyrimidinyl amino benzamido pentanoic acid as the most promising agent [34]. The synthesized compounds showed inhibitory activity comparable to C646. The most active compound, 4-(3-Cyclopropyl-4-((5-(4,5-dimethyl-2-(trifluoromethyl)phenyl)thiophen-2-yl)methylene)-5-oxo-4,5-dihydro-1*H*-pyrazol-1-yl) benzoic acid, showed IC_50_ = 0.16 µM, indicating inhibitory activity better than C646 with improved drug-like properties. Moreover, a greater reduction in H3K27 acetylation than C646 was confirmed by a Western blotting experiment. This compound also inhibited MCF7 and T47D with IC_50_ values of 22.54 µM and 5.08 µM according to a cellular assay [88].

Against two human cancer cells, MCF-7 and A549, the synthesized compounds exhibited a wide range of activities, having IC_50_ values 3.42–23.32 and 5.97–22.01 µM, respectively, compared with doxorubicin and erlotinib. The chloro anilinoquinoline derivative having IC_50_ values of 3.42 and 5.97 µM towards MCF-7 and A549 cancer cell lines, respectively, was the most active one [89]. Biological evaluation of four of the compounds as VEGFR-2 inhibitors exposed extraordinary inhibitory activity ranging from 83.4 to 87.3%. Accordingly, the compound cyano-hydroxy-methoxybenzylidene amino-phenyl-pyrrolyl-benzoic acid may be promising as an anticancer agent with an IC_50_ value 4-fold greater than vandetanib 2 [90]. The IC_50_ of compound *N*’-(3-chlorobenzoyl)-2-(2,4-dihydroxopyrimidine-5-sulfonamido) benzoyl hydrazide was 1.26 mM on A549 cells, which was much better than pemetrexed (PTX). According to flow cytometry analysis, the apoptosis rate was 39.4% for this compound, and it could effectively inhibit the proliferation of tumor cells [91]. Among the novel imidazo [4,5-*b*]pyridine derivatives, nine showed prominent activity against MCF-7, while remarkable activity against HCT116 was elicited for six compounds. Compounds with a remarkable CDK9 inhibitory effect had an IC_50_ range of 0.63–1.32 μM relative to sorafenib [33]. ((*E*)-N-(4-(1-(2-(4-benzamidobenzoyl)hydrazono)ethyl)phenyl)nicotinamide) inhibited VEGFR-2 with an IC_50_ = 51 nM and antiproliferative action against HCT 116 and MCF-7 cancer cells, having IC_50_ values of 6.48 µM and 8.25 µM, respectively [92]. Compounds depicted the maximum activities with 586.3, 636.2, and 705.7 pg/mL VEGFR-2 protein concentrations according to cytotoxic assessment. The docking results predicted that the synthesized compounds have the ability to identify the VEGFR-2 kinase ATP binding site and bind with amino acids like sorafenib [93].

The results concluded a moderate antiproliferative effect of all tested compounds against MDA-MB-231. Moreover, the 4-substituted coumarin, through 1,2,3-triazole linked with benzoyl 3,4-dimethoxyaniline, exhibited the most significant antiproliferative activities, having an IC_50_ value of 0.03 μM, about 20 times stronger than that of doxorubicin [94]. The antiproliferative activity of all compounds against five human cancer cell lines suggested remarkable cytotoxicity shown by Co(L)_2_Cl_2_ and Zn L)_2_Cl_2_ [82], and cisplatin was used as a reference drug. Molecular docking results were consonant with the experimental anticancer results. Excellent antitumor activity was shown for the MCF-7 cell line [95]. The cytotoxic activity of compound 4-(4-oxo-6-phenyl-2-thioxotetrahydropyrimidin-1(2*H*)-yl)benzoic acid was significant against cancer cell lines and had greater reactivity than doxorubicin [96].

A study of the activity of the malignant cell activity of a synthetic 1,3,4-thiadiazole derivative observed the effectiveness of the Schiff base of thiadiazole-1,3,4-(dimethylamino)benzylidineamino]-[4-2-phenyl]amino in decreasing the density and size and of malignant cells [97]. One of the synthetic compounds, hexadecyl 4-(4-oxo-2-phenylquinazolin-3(4*H*)-yl)benzoate, displayed notable cytotoxic activity with IC_50_ values of 23.31 ± 0.09 µM, 72.22 ± 0.14 µM, and 53.29 ± 0.25 µM against Caco-2, MCF-7, and HepG2 cancer cells, respectively [98]. Evaluation of anticancer activity against HepG2, MCF-7, and HCT-116 cells showed compounds that were the most active agents against cancer cell lines with IC_50_ values of 7.08 ± 1.6 to 10.87 ± 0.8 µM [99]. The results proved that thiazolidines-oxothiazolidinylidene)-amino-benzohydrazide and aminohydroxy-pyrazole-carbonyl-phenylimino-thiazolidinone have greater antibacterial as well as anticancer activity with IC_50_ = 45.32 ± 0.15 and 5.8 ± 0.24 μM against HCT-116 cell lines comparable with reference drugs [100].

## 5. Synthesis of Anti-Inflammatory Agents

**Scheme 4:**
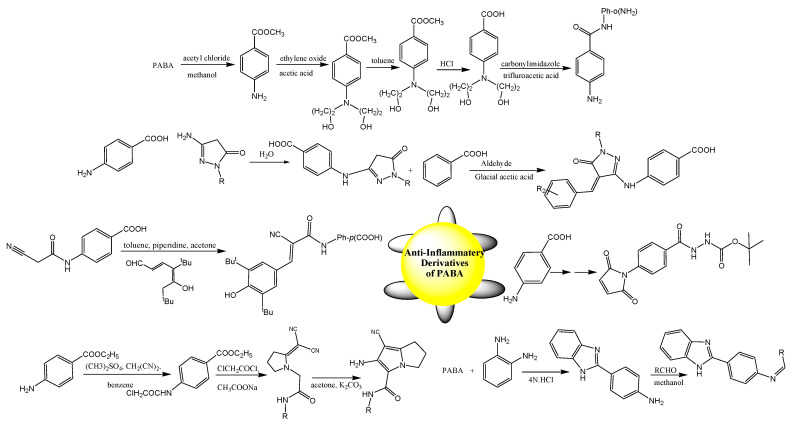
Synthetic routes of *p*-aminobenzoic acid derivatives having anti-inflammatory potential (adapted from refs. [102,103,104,106,107,108]).

A hydrazide derivative bearing two components, a maleimide and hydrazide moieties, was synthesized from *p*-aminobenzoic acid. Firstly, *p*-aminobenzoic acid was treated with maleic anhydride to obtain diacid. Cyclization was performed to give a targeted reported compound after the hydrolysis of a mixed anhydride intermediate. Lastly, an acid was activated with iso-butyl chloroformate and further treated with tertbutyl carbamate to obtain the required anti-inflammatory derivative [102]. 2-chloroethylamino benzamide was prepared using 4-aminobenzoic acid as starting material. Protection of PABA was provided by methyl esterification and benzoate-based moieties. Successively substituting chlorine with the carboxyl group and hydroxyl group and deprotecting resulted in intermediate 4-(bis(2-chloroethyl-based PABA, which, upon condensing with *o*-phenylenediamine, gave the target compound [103], as shown in Scheme 4. New pyrazolone derivatives were prepared that were structurally related to FPL 62,064 (5-lipoxygenase and COX dual inhibitor) and celecoxib. Key intermediates were first synthesized from 3-amino-pyrazol-5(4*H*)-one derivatives by refluxing with equimolar amounts of *p*-aminobenzoic acid in water and hydrochloric acid. Arylidene derivatives were synthesized by reacting pyrazolone derivatives with aromatic aldehydes in dry sodium acetate [104]. The peptide derivatives having N_3_ and SO_2_Me functional groups at the *para* position were bound to various aromatic amino acids. Similarly, peptides possessing an azide group were prepared by the first reaction of PABA with NaN_3_ and NaNO_2_. This azido acid was attached to peptide-resin, and the final peptide was cleaved from resin, giving a product having carboxyl and N_3_ or SO_2_Me functionalities [105]. A mixture of 2-pyrrolidin-2-ylidenemalononitrile, ethyl 4-(2-chloroacetamido)benzoate, and anhydrous K_2_CO_3_ in anhydrous acetone was stirred under reflux for 24 h, resulting in the synthesis of ethyl 4-(6-amino-7-cyano-2,3-dihydro-1*H*-pyrrolizine-5-carboxamido)-benzoate. Benzimidazole-based Schiff bases were obtained as 2-(4-aminophenyl)benzimidazole by refluxing *p*-aminobenzoic acid with *o*-phenylenediamine in an acidic medium, which, upon reaction with aromatic aldehydes under reflux in methanol, gave the resultant 2-substituted benzimidazole-based Schiff bases [106] (Scheme 4). The synthesis of novel PABA was achieved by condensing cyanoacetyl aminobenzoic acids with the tertbutylhydroxybenzaldehyde [107]. Scheme 4 shows the routes for the synthesis of PABA derivatives having anti-inflammatory potential.

### Anti-Inflammatory Activities

Data from in vitro anti-inflammatory and antiproliferative activity disclosed that, among the molecules under study, the derivative of PABA (DAB-2-28) possesses lesser cytotoxic activity with higher efficiency [102]. An enzymatic activity inhibition assay performance against compounds unveiled that the benzamide-derived compound showed good results with IC_50_ values of 95.2, 260.7, and 255.7 nM against histone deacetylases (HDAC1, HDAC2, and HDAC3, respectively), while its antiproliferative assay expressed an IC_50_ of 2.66 μM (10.3 times) and 1.73 μM (11.3 times), showing greater efficacy than suberoylanilide hydroxamic acid [103]. Biological evaluation against cyclooxygenases (COXs) and 5-lipoxygenase (5-LOX) inhibition and their selectivity indices exhibited that four compounds have an outstanding COX-2 selectivity index. Moreover, potent 5-LOX inhibitory activity comparable to celecoxib and zileuton was also observed [104]. The evaluation of in vitro cyclooxygenase (COX) inhibitory action exhibited higher selectivity of benzoate-derived compounds to COX-2 than to COX-1, with a selectivity index value of 3.46 [108]. Substantial scavenging activity with ascorbic acid as a standard was determined for test compounds. Furthermore, compounds had scavenging activity greater than 75%. Noticeable analgesic and anti-inflammatory effects were seen in some of the targeted benzimidazole Schiff bases [106]. The phenylacrylamide core-based compounds with butyl-4-hydroxy group on a phenyl ring and benzoic acid were vital for drug-likeness properties as well as for bioactivity as nuclear receptor ligands [107].

## 6. Synthesis of Antiviral Agents

**Scheme 5:**
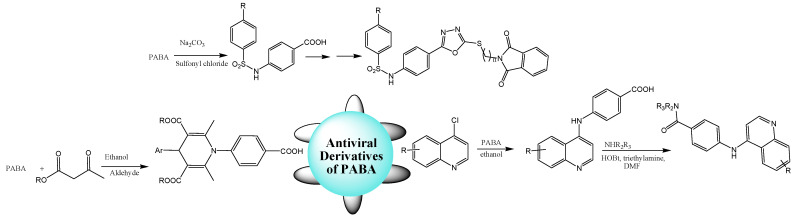
Synthetic routes of derivatives of *p*-aminobenzoic acid having antiviral potential (adapted from refs. [109,110,111]).

The substituted pyridine ring was synthesized by mixing alkyl acetoacetate, properaldehyde, and para-aminobenzoic acid. The mixture was refluxed in absolute ethanol to yield arylpyridinyl benzoic acid derivatives [109]. Quinolinyl aminobenzoic acids were prepared by a reaction of 4-chloroquinoline derivates with *p*-aminobenzoic acid by refluxing the mixture in ethanol. Desired quinoline analogs were synthesized by condensing quinolinyl-substituted aminobenzoic acid and amines [110] as shown in Scheme 5. The synthesis of innovative *S*-alkylphthalimidated and *S*-benzylated hybrids was achieved. With substituted benzenesulfonyl chlorides, *p*-aminobenzoic acid was reacted through its amino group for the first series of hybrids, whereas the other carboxylic acid side was converted to sulfonamido 1,3,4-oxadiazole-2-thiols. The resulting intermediates were further transformed into *S*-alkylphthalimidated or *S*-benzylated hybrids by reaction with halides. For another series of hybrids, the conversion of the carboxylic acid of probenecid to sulfonamido-1,3,4-oxadiazolethiols and then to *S*-alkylphthalimidated and *S*-benzylated hybrids was performed [111]. The synthetic routes for the synthesis of derivatives of PABA having antiviral potential are shown in Scheme 5.

### Antiviral Activities

An intermediate to excellent inhibition against HIV proliferation was exhibited by most of the compounds, and cytotoxic activity was also observed. Compounds showed effective anti-HIV action at 100 μm given the percentages of inhibition (84–76%). Docking studies confirmed the compound styryl pyridinyl benzoic acid as the most potent one having static and hydrophobic bonding contacts with a gp41 active site [109]. For the evaluation of the anti-influenza activity of tested compounds, inhibition, cytotoxicity, and cytopathic assays were also carried out. The targeted compound benzamide possesses significant anti-influenza virus activity with both inhibition and cytopathic activity having IC_50_ = 0.23 ± 0.15 µM and EC_50_ = 11.38 ± 1.89 µM, respectively [110]. Significant activity against various influenza virus strains was also observed. According to bioactivity assays, benzenesulfonamides are the most powerful inhibitors, having IC_50_ values of 13.9 μΜ and 15.1 μΜ [111]. Table 1 summarizes the data from various research articles related to the therapeutic potential of PABA derivatives.

## 7. Future Perspectives and Challenges

Para-aminobenzoic acid is a naturally occurring chemical that is commonly employed as a dietary supplement and possesses a range of potential physiological advantages. PABA analogs have been discovered to possess protective properties against ultraviolet (UV) radiation, a known factor that can induce skin damage and accelerate the aging process. Subsequent investigations may delve into the prospective application of said substance as an organic sunblock or as an ingredient in skincare formulations. Additional research could investigate the use of PABA as an ingredient in hair care formulations, as it has demonstrated significant advantages for promoting hair well-being, such as the mitigation of premature greying and hair loss. Para-aminobenzoic acid analogs’ synthesis and modification have made it possible to design new compounds with better pharmacological profiles and fewer adverse effects. Furthermore, these analogs are promising candidates for targeted therapeutics since the mechanisms of action describe that they interact with certain molecular targets. There are interesting possibilities for new medication development to inhibit the enzymes, stop DNA replication, or regulate immunological responses. It is also important to consider the toxicity, bioavailability, and stability of PABA analogs. To confirm their efficacy and safety characteristics, thorough preclinical and clinical investigations are also required.

Additionally, further exploration of PABA’s potential anticancer effects in animal studies could be pursued. These substances have the potential to completely alter the field of drug development and therapies with further study and innovation. These analogs may play a significant role in the future of personalized and precision medicine by providing treatments that specifically fulfill the needs of each patient. Validating the efficacy and safety of PABA analogs will mostly depend on increasing clinical studies, assuring long-term safety evaluations, and researching synergistic combinations of PABA analogs with other medications.

## 8. Conclusions

Para-aminobenzoic acid has dual functionalities, due to which it has great potential in organic synthesis and medicinal chemistry. It is being used as a substitute for many other important building blocks of naturally occurring compounds. Researchers are being engaged in exploring the use of PABA as a dietary supplement or as an ingredient in functional foods, as it has been shown to have antioxidant properties that protect against cellular damage. The derivatives of PABA have the potential to cure cancer and neurorelated diseases. Although they have great potential to treat various diseases related to human beings or animals, the toxicity consequences associated with PABA analogs are the most significant challenge. In preclinical tests, certain analogs showed cytotoxicity, raising questions about their long-term safety in human patients. To reduce these hazards, rigorous toxicity evaluations and dosage optimization are required. Another difficulty is attaining adequate bioavailability. Many PABA analogs have low solubility and absorption in the human body, which can reduce their efficacy. Furthermore, when PABA analogs are used for extended periods of time, particularly in antimicrobial therapy, resistance mechanisms might evolve. Identifying and tackling these resistance mechanisms is critical to ensuring the long-term efficacy of these medicines. There is a need in this era to conduct detailed research on the mechanism of action of PABA and its analogs, along with various modifications through which we can reduce its site effect and increase its efficacy.

## Figures and Tables

**Table 1:** Synthesis and reaction conditions related to therapeutic potential of *p*-aminobenzoic acid derivatives.

Derivatives of PABA	Synthetic Conditions	Characterization Techniques	Physical Properties	Biological Activity	IC_50_ (µM)	Reference
Nonyl 4-nonylamino)benzoate	PABA, alkylating agent, K_2_CO_3,_ acetone, 5–6 h	EIMS, FTIR, ^1^H-NMR, and ^13^C NMR	Amorphous white solid	Cytotoxicity activity against the NCI-H460 cell line	NCI-H460: 15.59	[112]
4-((5,6,7,8-tetrahydrobenzo [4,5]thieno [2,3-d]pyrimidin-4-yl)amino)benzoic acid	4-chloro-6,7,8,9-tetrahydro-5H-indeno [2,1-d]pyrimidine, PABA, ethanol, reflux, 4 h	EIMS, FTIR, ^1^H NMR, and ^13^C NMR	Yellow crystal, Yield: 88%, m.p: 280–282 °C	Cell lines: EGFRT790M and HER2 kinases and 4 cancer cell lines of HCT-116, HepG2, A431, and MCF-7	HepG2: 67.72 ± 3.70 HCT-116: 62.57 ± 3.30 MCF-7: 75.75 ± 3.40	[113]
*N*-(tert-butyl)-4-(2-(3-methyl-2-oxoquinoxalin-1(2H)-yl)acetamido)benzamide	*N*-(tert-butyl)-4-(2-chloroacetamido) benzamide, potassium salt of 3-methylquinoxalin-2(1H)-one, DMF, KI, heating 3 h	^1^H NMR, ^13^C NMR, and FTIR	Dark green crystal, Yield: 80%, m.p: 230–232 °C	Cytotoxicity against HepG2 and MCF-7 cell lines	MCF-7: 7.7, HepG2: 4.5	[114]
2-(4-((2-Oxoindolin-3-ylidene)amino)benzoyl)-*N*-phenylhydrazinecarbothioamide	4-((5-Substituted-2-oxoindolin-3-ylidene)amino)benzohydrazide, Isothiocyanatobenzene, EtOH, Reflux, 8 h.	EIMS, FTIR, ^1^H NMR, and ^13^C NMR	Yellow powder, Yield: 93%, m.p: 211–213 °C	Cytotoxic activity against HepG2 cell line, MCF-7 cell line, VEGFR-2 kinase inhibitory assay	MCF-7: 0.74–4.62 HepG2: 1.13–8.81 VEGFR-2: 0.078	[19]
((*E*)-*N*-(3-(1-(2-(4-(2,2,2-Trifluoroacetamido)benzoyl)hydrazono)ethyl)phenyl)nicotinamide)	2,2,2-Trifluoro-*N*-(4-(hydrazinecarbonyl)phenyl)acetamide, *N*-(3-acetylphenyl)nicotinamide, 6 h and absolute ethanol	EIMS, FTIR, ^1^H NMR, and ^13^C NMR	Off-white crystals Yield: 80%, m.p: 246–248 °C	Cytotoxic properties against VEGFR-2, MCF-7 cell lines, and HepG2	VEGFR-2: 65 nM HepG2: 21.00, MCF-7: 26.10	[115]
3*α*-O-(4-acrylamidebenzoyl)-4β-hydroxy-5,7α,6β(H)-guaia-1(10),11(13)-dien-12,6-olide	3*α*,4*β*-dihydroxy-5,7*α*,6*β*(H)-guaia-1(10),11(13)-dien12,6-olide, 4-acrylamidobenzoic acid, DCC, DMAP, CH_2_Cl_2_,	^1^H NMR, ^13^C NMR, and HRESIMS	White powder, Yield: 56%, m.p: 120–122 °C	Cytotoxicity against Huh7 and HepG2 cell lines	HepG2: 7.9 ± 0.3 Huh: 78.5 ± 0.2	[116]
Ag-PABA complex	PABA, KOH, AgNO_3_, 1 h	XRD, mass spectrometry, ^1^H NMR, 2D NMR, and ^13^C NMR	White solid	In vitro antibacterial activity	MIC value (µM) *S. aureus* ATCC 25923: 88.5 *B. cereus* ATCC 14579: 88.5 *E. coli*: 44.0	[117]
4-[1-(1,3-Dihydro-benzoimida-zol-2-ylideneamino)-oct-2-ynylideneamino]-benzoic acid	Oct-2-ynoic acid (1,3-dihydrobenzoimidazole-2-ylidene)amide, aniline derivatives, warm ethanoic solution and refluxing for 4 h	UV–Vis absorption, FTIR, ^13^C NMR, ^1^H NMR, and C, H, N elemental analysis	White powder, Yield: 73%, m.p. 175.2 °C	Anti-plasmodial activity	*Plasmodium Falciparum* 3D7: 28.31 µg/mL	[118]
Peanut skin extract-azo-compounds and complexes with iron	NaNO_2_, HCl, ice, peanut skin extract, FeCl_2_, methanol, 65 °C, reflux 3 h	SEM-EDS analysis, FTIR, and UV-visible spectroscopy	Deep orange, Yield: 56.81%, m.p: > 300 °C	Antimicrobial activity against *Staphylococcus aureus* and *Escherichia coli*	Inhibition zone (mm) *E. Coli:*10.0 ± 0.33 *S. aureus:* 6.0 ± 1.52	[119]
4-(1H-Indole-2-carboxamido)benzoic acid	Dry pyridine, CHCl_3_,1H-Indole-2-carbonyl chloride	NMR, IR spectroscopy, HRMS, and elemental analysis techniques	Yield: 86%, m.p: 256–258 °C	Antifungal and antibacterial activity	Inhibition zone (mm) *C. albicans* DSMZ 11949: 9–14	[120]
Methyl (S)-2-(4-(4-methylphenylsulfonamido)benzamido)propanoate	Amino acid methyl ester hydrochloride, 4-(4-methylphenylsulfonamido)benzoic acid, DCC, HOBt, DIPEA, THF,	^1^H NMR, ^13^C NMR, and HRMS	Yield: 92%, m.p. 203.1–203.9 °C	In vitro PPRE-activated activity	PPRE-activated activity: 87%	[27]
2-((4-(*N*-(4-Carboxyphenyl)sulfamoyl)phenyl)carbamoyl)benzoic acid	2-((4-(*N*-(4-(Ethoxycarbonyl)phenyl)sulfamoyl)phenyl)carbamoyl)benzoic acid, NaOH, EtOH, 80 °C, 18 h	^13^C NMR, ^1^H NMR, and HRMS (ESI)	White powder, Yield: 82%, m.p. 203–205 °C	Antiviral activity against Nancy, coxsackievirus B3. CVB3	CVB3: 4.22 Nancy: 4.29	[121]
*N,N’*-(1,2-phenylene) bis(4-aminobenzamide) and its complexes	ethyl 4-aminobenzoate, 1,2-Benzene diamine, reflux 3 h, MCl_2_, reflux	UV-Vis, FTIR, ^1^H NMR, and elemental analyses	Pale green, Yield: 45%, m.p: 230 °C	Antibacterial activity against *S. aureus* and *E. coli*	Diameters of antibacterial activity *S. aureus*: 25 mm *E. coli*: 28 mm	[122]
*N*-(2-(1-benzylpiperidin-4-yl)ethyl)-4-((3,5-dimethoxybenzyl)amino)benzamide	*4*-Amino-*N*-(2-(1-benzylpiperidin-4-yl)ethyl)benzamide*,* benzyl halide ACN, 80 °C, 6 h,	^13^C NMR, ^1^H NMR, and HRMS (ESI)	Yellow solid, Yield: 80%, m.p: 118–120 °C	Activity against ChE enzymes	AChE: 0.61, BchE: 2.04	[123]
*N*-(4-{[(2E)-2-(4-(2,3,4,6-tetra-O-acetyl-β-D-galactopyranoside)-3-metoxybenzylidene)hydrazino]carbonyl}phenyl)benzamide	galactoside, *N*-(4-(hydrazinecarbonyl)phenyl)benzamide, ethyl alcohol, r.t, HCl, stirring for 24 h	^13^C NMR, ^1^H NMR HRMS (ESI), and FTIR	White crystals, Yield: 84%, m.p: 137–138 °C	Antifungal activity	*C. glabrata*: 695.2	[124]
4-(4-(1-(2-Isonicotinoylhydrazono)ethyl)phenylsulfonamido)benzoic acid	Acetylbenzenesulfonamide derivatives, hydrazide derivatives, methanol, 2 h, glacial acetic acid	UV, IR, ^1^H NMR, ^13^C NMR, mass spectroscopy, and elemental analysis	White solid, Yield: 41% m.p: 288–290 °C	AChE activity	AChE: 11.12 ± 0.74	[125]
*N*-(3-(Hydroxycarbamoyl)phenyl)-3,5,6-Trimethylpyrazine-2-Carboxamide	4-(3,5,6-trimethylpyrazine-2-carboxamido)benzoic acid, NH_2_OH,	^1^H NMR	Yield: 52% m.p: >250 °C	Antiproliferative activities against two HDAC-expressing cancer cell lines: HT-29 and SH-SY5Y	HT-29 cells: 1.96	[126]
Cu(PABA)_2_(H_2_O)_2_ complex	Metal and ligand 1:2 molar ratio (M:L), 30 min, pH value at 5.6, r.t	FTIR and UV-vis spectroscopy	Dark green product	Antimicrobial activity	Inhibition zone (mm) *Enterococcus faecalis:* 16 *S. aureus*: 11	[14]
4-(2-(4-(1H-Benzimidazol-2-yl)phenyl)hydrazono)-1-(4-chlorophenyl)-3-methyl-1H-pyrazol-5(4*H*)-one	Ethyl 2-(2-(4-(1H-benzimidazol-2-yl)phenyl) hydrazono)-3-oxobutanoate, hydrazine hydrochloride, ethanol, refluxed for 12 h	EIMS, FTIR, ^1^H NMR, and ^13^C NMR	Yield: 82%, m.p: 197–200 °C	Antimicrobial activity	MIC (µg/mL) *S. aureus*: 7.81 *S. epidermidis:* 1.95 *E. coli*: 7.81	[127]

## Data Availability

Data is available on request from corresponding author.
